# Classification of Low Frequency Signals Emitted by Power Transformers Using Sensors and Machine Learning Methods

**DOI:** 10.3390/s19224909

**Published:** 2019-11-10

**Authors:** Daniel Jancarczyk, Marcin Bernaś, Tomasz Boczar

**Affiliations:** 1 Department of Computer Science and Automatics, University of Bielsko-Biala, 43-309 Bielsko-Biala, Poland; djancarczyk@ath.bielsko.pl; 2 Institute of Electric Power Engineering and Renewable Energy, Opole University of Technology, 45-758 Opole, Poland; t.boczar@po.opole.pl

**Keywords:** low-frequency sensor, power transformer, machine learning, low-frequency noise, classification

## Abstract

This paper proposes a method of automatically detecting and classifying low frequency noise generated by power transformers using sensors and dedicated machine learning algorithms. The method applies the frequency spectra of sound pressure levels generated during operation by transformers in a real environment. The spectra frequency interval and its resolution are automatically optimized for the selected machine learning algorithm. Various machine learning algorithms, optimization techniques, and transformer types were researched: two indoor type transformers from Schneider Electric and two overhead type transformers manufactured by ABB. As a result, a method was proposed that provides a way in which inspections of working transformers (from background) and their type can be performed with an accuracy of over 97%, based on the generated low-frequency noise. The application of the proposed preprocessing stage increased the accuracy of this method by 10%. Additionally, machine learning algorithms were selected which offer robust solutions (with the highest accuracy) for noise classification.

## 1. Introduction

The operation of medium- and high-power transformers is associated with low frequency noise emission into the environment, the main source of which, among other things, include: cooler fans of the induced air circulation, insulation oil circulating pumps, and the magneto-strictive vibrations of the core. In very simple terms, magnetostriction means that if a piece of magnetic sheet steel is magnetized, it extends. When the source of magnetization is removed, it goes back to its original state. A transformer is magnetically excited by alternating voltage and current so that it extends and contracts twice during a full cycle of magnetization. 

Identifying the problem of low frequency noise generated by new and worn out power transformers requires a wide range of tests. The reference measurement methodologies, methods of analysis, and the assessment of the nuisance caused by the generated noise should be clearly defined. Power transformers are considered as strong sources of low-frequency noise, with the most important spectrum components, in view of the noise level, being in the frequency range below 400 Hz. The initial results obtained in the experimental tests and measurements on real units can be referenced to the maximum allowable low frequency noise levels, which are defined in the standards relating to the working environment. Therefore, it is vital to be able to detect the low frequencies of power transformers and their levels (specificity). 

The aim of this paper is to propose a method for the automatic detection of noise generated by a power transformer. The method makes it possible not only to detect the presence of noise from a transformer in the area, but also to determine the transformer type, its internal construction, and apparent power. Furthermore, the type of misclassification (i.e. detection of a transformer with higher apparent power) can be used as an initial diagnostic tool that detects the changing parameters of the device. It is worth noting that low-noise measurements can be made during the normal operation of the transformer (online).

The proposed method involves data acquisition using a dedicated low frequency sensor, a preprocessing stage (applying frequency analysis), and a classification stage using machine learning algorithms.

The scope of the analysis reported here includes the determination of waveforms showing changes in the sound pressure level as a function of frequency (amplitude spectra). For comparative purposes, the characteristics were determined separately for the examined transformers and background noise at the selected measurement points.

The paper is organized as follows. Section 2 describes previous works related to the low frequency sound analysis for transformers and machine learning methods. The proposed method for transformer detection based on sound pressure is presented in Section 3. Section 4 includes the experimental results and a comparison of the introduced method with state-of-the-art approaches. The conclusions and future research directions are given in Section 5.

## 2. Related Works

Infrasound noise is taken to be called noise, in which the spectrum is dominated by low frequencies, i.e., up to 20 Hz, in accordance with the PN-ISO 7196: 2002 standard [1]. There is no standardization of low frequency sounds; however, most scientists consider such frequencies to include the range between 10 and 200 Hz [2]. 

As part of the initial research, an attempt was made to determine the emission level of low-frequency signals generated by power transformers at rated conditions. The distribution transformers reducing the voltage from 15 kV to 0.4 kV of various types (indoor and overhead) and apparent powers (400 kVA and 2000 kVA) were tested [3]. 

The research conducted to date has demonstrated that power transformers are a source of low frequency signals. Our studies showed that they are characterized by similar waveforms of averaged amplitude spectra in terms of shape, as well as a similar character of time-frequency changes. The waveforms characteristic of the recorded sound pressure level has dynamically decreasing values, which occur within a frequency range of 10 Hz to 100 Hz [3,4]. In the conducted research, the Brüel & Kjær measuring equipment was applied, focusing only on the measurement of the sound pressure level without converting it to the sound power level. This approach can be effectively applied to determine the range of potential impact of low-frequency sound directly at the location of the measuring point.

Similar research was conducted for wind turbines, which are also covered by a wide range of research on infrasound and low-frequency noise [2]. Usually, the scope of the analysis includes the development of curves such as hysteresis that present variations in parameters over time, and the designation of the frequency spectra of the recorded low frequency signals for different meteorological conditions, often including wind speeds and direction. Usually, the sound pressure level of infrasonic noise for a given wind speed is determined as an arithmetic mean from all recorded sound pressure levels for the speed. A commonly used method for spectrogram analysis is the short time Fourier transform (STFT). The next step in the research study involved the analysis of the frequency spectra of sound pressure levels corrected with G frequency characteristics, which can be employed in demonstrating noise levels in conditions that are audible to the human ear [5,6]. Other studies present the effect of infrasound noise related to everyday human activities [7].

To date, measurements and signal analyses of power transformers were conducted by experts. This paper proposes the automation of a process involving the detection of operational transformers in the vicinity and their classification using Machine Learning algorithms (ML), which makes it possible to find complex relations and rules via data mining techniques. ML roots can be found in pattern recognition and computational learning theory. The method uses learning algorithms and example data (training set) to build the model, which can be adopted for classification or prediction purposes. There are three main categories of learning algorithms: supervised, unsupervised, and reinforced. In the case of supervised learning [8], a training set is given with the correct target values. In case of unsupervised learning [9], we tend to find the relation in some given data without knowing its original (correct) classification. Reinforcement learning [10] solves optimization problems, learning the optimal actions for a given situation. This paper is focused on supervised learning, due to the characteristics of the processed data. The classification algorithms commonly found in the literature includes: k-Nearest Neighbors [11], Naive Bayes Classification [12], Support Vector Machine [13,14], Random Forests [15], Bagging [16], and various types of Neural networks [17,18,19,20].

One of the major objectives in ML is to identify the useful internal representation of the input data by means of preprocessing methods, which transfer them into a new variable space. Preprocessing can simplify the model and improve its accuracy. However, in this case, a deeper understanding of the researched phenomenon is needed (i.e., a heuristics-based approach) to find the optimal feature representation [9] corresponding to the analyzed data. Commonly applied preprocessing methods include Principal Component Analysis [21,22,23,24] and Canonical Correlation Analysis [25,26].

It is worth noting that Neural Networks (especially deep ones), due to their specificity, find applications in both classification and pattern recognition. This approach overcomes the shortcomings of linear models by allowing a defined number of basic function parameters to learn within a defined network. There are many different types of neural networks (distinct by neurons construction and organization). They are easily adaptable to regression and classification problems [17,18]. Feed Forward Neural Networks (FFNN), also known as Multilayer Perceptron (MLP), are the most common type of neural networks. Based on the number of hidden layers, the network can solve more complex, non-linear problems. Unfortunately, they require a large amount of computation for their training (based on network complexity). Finding the optimum set of weights in cases of multiple hidden layer structures is a NP-complete problem [27]. Therefore, an alternative for more than one hidden layer MLP networks was proposed (deep neural network), in which layers have their functions, i.e., analyses of higher-level features based on the low-level features of previous layers [17,20]. Significant results using deep neural networks have led them becoming the most common classifiers in machine learning [27,28].

Previously presented ML algorithms have been proposed for sets of data that are independent. However, in the considered case, the measured time series contains sound pressure values of a defined period of time which is characterized by significant sequential correlations [9,29], and should be represented as a temporal feature. This task can be performed by sequence classification, in which the sequences of time series are taken into consideration [29]. There are a variety of machine learning models and methods that can perform these tasks. Examples of these models and methods appear under the names of Markov models [30], sliding-window methods [31], Kalman filters [32], conditional random fields [33], recurrent neural networks [34], graph transformer networks [17], the Welch method, and maximum entropy Markov models [35]. Further analysis can be found in [36]. The Welch method [37] makes it possible to determine the estimated spectral power density of the signal. As research shows [37], this method can be employed to minimize the effect of external noise, by averaging/smoothing the instantaneous spectrum. Additionally, it can be applied to identify the frequencies that could contain useful information for classification purposes. Therefore, in the proposed model, the Welsh method will be used in the preprocessing stage with the proposed feature discrimination method and various ML algorithms. 

## 3. Methodology and Characteristics of the Analyzed Power Transformers 

**Figure 1:**
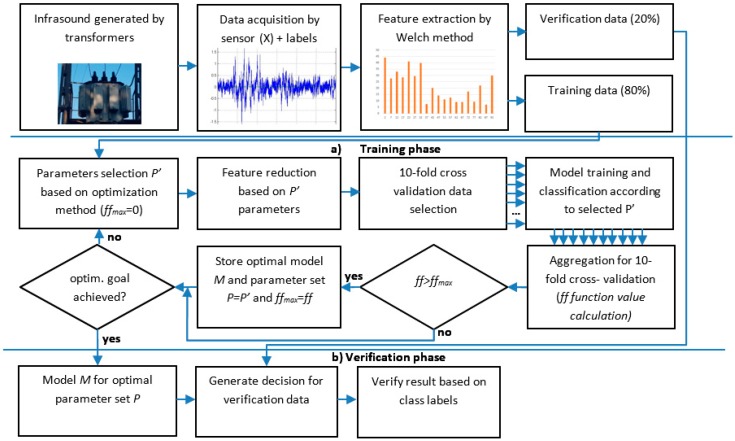
Overview of proposed method.

Based on previous research concerned with the transformer infrasound noise [1,3], it was observed that noise could be detected in all frequency spectra, but that some frequencies were found to be dominant. On the basis of this observation, a method was proposed to find the optimal frequency intervals and automatically detect noise using a selected machine learning algorithm. An algorithm providing the overview of this method is presented in Figure 1. 

In a first step, the low frequency sound generated by the transformer or background is registered using a dedicated sensor device. The data are represented as time series *X* = [*x*_1_, *x*_2_, …, *x*_n_], where *n* is the number of samples. To reduce the volume of data in time series *X*, it is transformed into frequency domain vector *F* by the application of the Welch method. Then, using parameters *P* = [*l*, *h*, *s*, *m*_1_, *m*_2_, …, *m_k_*], the model is tuned. The parameters *l* = [2, 100], *h* = [2, 100], and *l < h* defines respectively lower and upper frequency bands, s∈N influences sample resolution, and *m_i_*, where *i* = 1, …, *k*, defines the ML algorithm parameters. In the first phase of the method (Figure 1a), initial parameters *P’* are selected. The vector *F* size is reduced using *l, h,* and *s* parameters, and then it is used (together with class labels) to train the ML (tuned by *m_x_* parameters) using ten-fold cross-validation to increase the soundness of the result. The obtained classification accuracy (*acc*) is used to calculate fitness function *ff* (Equation (1)).

(1) $$ff=acc-\frac{h-l}{s\times 10000},$$

where $acc\;=\;\frac{TP+TN}{TP+TN+FP+FN},$ TP is the true positive (correct classification), TN is the true negative (correct rejection), FP is false positive (type I error), and FN is false negative (type II error).

The proposed fitness function ensures that the result with highest accuracy will be selected, while results with fewer samples will be favored in cases when a comparable level of accuracy level is gained.

The number of iterations depends on the chosen optimization method. This research involved exhaustive search and heuristic methods. The parameters for the iteration with the highest *ff* function value and generated model *M* are used in the verification phase to estimate the model’s robustness. 

Several optimization algorithms and ML methods were analyzed to find the optimal solution. In this section, all the used algorithms are described and discussed.

### 3.1. The Data Acquisition Method

**Figure 2:**
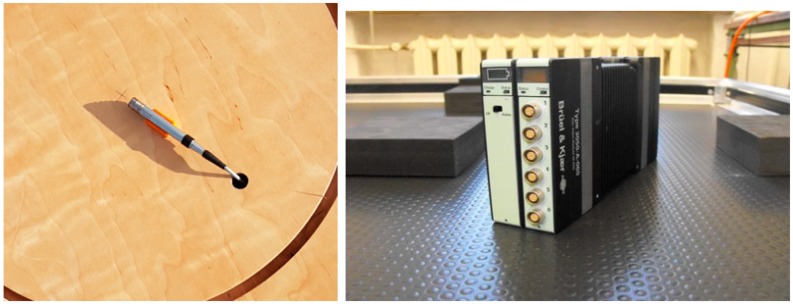
Microphone type 4190 digital and signal meter with LAN-XI type 3050-A-60.

The sensor device comprises a microphone type 4190, designed for accurate free field measurements, connected to a preamplifier type 2669L from Brüel & Kjær (Nærum, Denmark) and a digital signal meter with registration function LAN-XI type 3050-A-60, also from Brüel & Kjær. It was used to register low frequency signals, as shown in Figure 2. It is a professional tool used to measure sound pressure, intensity, and vibrations. Its implementation possibilities are wide ranging, i.e., from typical acoustic tests, such as noise measurements, the determination of sound power levels, noise mapping using beamforming techniques, testing the acoustic properties of materials, and determining the acoustic parameters of rooms, to specialist acoustic tests, such as machine diagnostics, modal analyses, and electroacoustic tests of acoustic transducers [2]. In the case of the used set, the range of the measured frequencies varied from 0.7 Hz to 20 kHz.

Before starting the measurements, the system was calibrated using the Brüel & Kjær type 4231 acoustic calibrator. 

**Figure 3:**
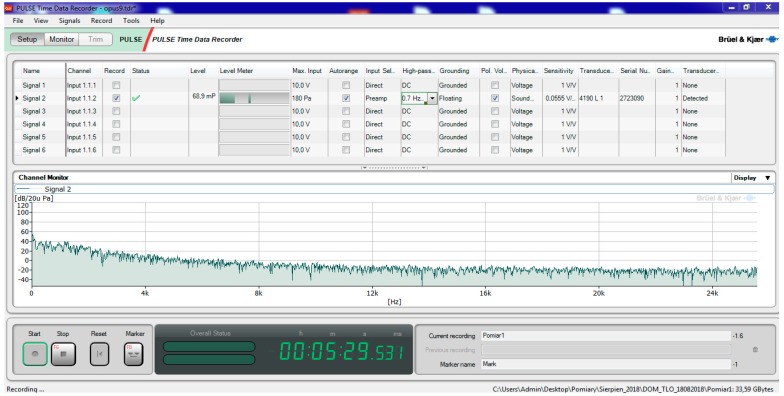
PULSE LabShop application screenshot.

To operate the meter, the computer was used with dedicated software which was connected to a LAN cable measuring system. All operating parameters were defined using the PULSE LabShop application version 15.1.0, which forms an integral part of the setup shown in Figure 3. Apart from the option of the precise configuration of the device, this software provides tools with which to record measured signals and preprocess and visualize them in offline mode. This is a dedicated software package developed by Brüel & Kjær Sound & Vibration Measurement A/S.

The measurement was carried out through a continuous, multi-hour process using a sampling frequency of 51.2 kHz (full registration with listening capability). In addition, all analyzed power transformers were located away from major roads and motorways.

### 3.2. Feature Extraction and Optimization Methods

The feature extraction from time series *X* was performed using fast Fourier transform with a Hamming window. The procedure is called the Welch method [37]; it allowed us to determine the estimated spectral power density of the signal. The method aims to minimize the influence of external noise by averaging/smoothing the instantaneous spectrum. The parameters of the method were adapted to suit the characteristics of the test apparatus and the analyzed frequency range [2]. 

As a result, the vector *F* = [*f*_1_, *f*_2_, …, *f_n_*] was generated, which defines the values of sound pressure for a given frequency, in this case, a low frequency (2–100 Hz). The *n* value depends on the resolution of transformation. In this case, *df* = 0.125 Hz, yielding n = 784 values. To decrease quantity of input data, its resolution can be modified by the *s*, s∈N. parameter. The *F’* vector was defined using the *s* parameter value (Equation (2)):

(2) $$F^{\prime }=\left[f_{1}^{\prime },f_{2}^{\prime },\ldots ,f_{i}^{\prime },\ldots ,f_{\left[\frac{n}{s}\right]}^{\prime }\right],f_{i}^{\prime }=f_{\left(i-1\right)*s+1}^{},f_{i}^{}\in F.$$

For instance, for s = 8, the reduced vector stores only values representing data for a 1 Hz resolution (99 features). Finally, the vector *F’* is further reduced, and it contains only values for a given frequency interval. The operation is performed using two filters tuned by the lower (*l* ∈N) and upper band (*h* ∈N) parameters. As a result, the final vector of features is generated (*F*″) (Equation (3)):

(3) $$F^{\prime \prime }=\left[f_{1}^{\prime \prime },f_{2}^{\prime \prime },\ldots ,f_{j}^{\prime \prime },\ldots ,f_{[\frac{h-l}{df*s}]+1}^{\prime \prime }\right],f_{j}^{\prime \prime }=f_{\left[\frac{l-2}{df*s}+j\right]}^{\prime },f_{}^{\prime }\in F\prime$$

which is used as an input for the ML algorithm. It is worth noting that *l*, *h*, and *s* are part of *P* parameter vector. 

The search for optimal *P* vector values was performed using various optimization methods. Due to the small intervals of the analyzed frequency, it was possible to use an exhaustive method (EM) that made it possible to search through all the available parameter regions to find the optimal one. Nevertheless, several additional methods were also researched. The first is called Hill Climbing (HC), and starts with random parameter values. Then, the optimal solution was searched for among the surrounding values. If no neighbors improved the result, the optimization procedure was terminated. The second one, called random search (RS), also selects the initial parameters randomly, then the next position is selected randomly within the search space. The algorithm ends after the definition of 1000 steps or if the result does not improve over 20 steps. Finally, the Bayesian optimization (TPE) strategy was used which consists of two phases; the first is the warm-up, in which parameter combinations are randomly selected and evaluated. Based on the scores of the warm-up rounds, the second phase tries to find promising parameter combinations, which are then evaluated [38]. 

### 3.3. Machine Learning Model (ML)

The machine learning model M was designed and developed using reduced training data set *F*″ and parameters *m_x_*. The various ML algorithms were researched to check their applicability for the purposes of this task. They include k-Nearest Neighbors (KNN), Multilayer Perceptrons network (MLP), Classical Support Vector Machines (SVMs), and the Bayes approach. The implementation of these methods was based on the Weka library [39]. The KNN method classifies a new data vector by looking at the *k* given vectors that are closest to it in the feature space. In the proposed method, Euclidean distance, with *k* = *m*_1_ as a parameter, was selected. For the case of probabilistic classifiers, we can apply those that identify the naive Bayes family on the basis of Bayes’ theorem with the assumption of independence among the features; thus, no parameters are required. The SVMs non-probabilistic approach aims to identify hyperplanes that separate search classes. In this research, linear SVM was used that finds a hyperplane that is a linear function of the input features. Several parameters, like normal vector to the hyperplane by *w* and the parameter for controlling the offset *b*, as well as variable ξi, were preset based on research reported in [13,14]. For the random forests method, where instead of training a single tree, many trees are trained, the number of trees was defined as *t* = *m*_1_ parameter. Based on the initial research, the maximal number of trees was set to 10. Above this level, no improvement in accuracy was recorded. The MLP was selected to be representative of an artificial neural network. It proved adequate for the purposes of classification tasks. In this case, ≤ 2 hidden-layer (*L* = *m*_1_) and ≤ 20-neurons-per-layer (*m*_2_) structures were considered. 

## 4. Results and Discussion

**Figure 4:**
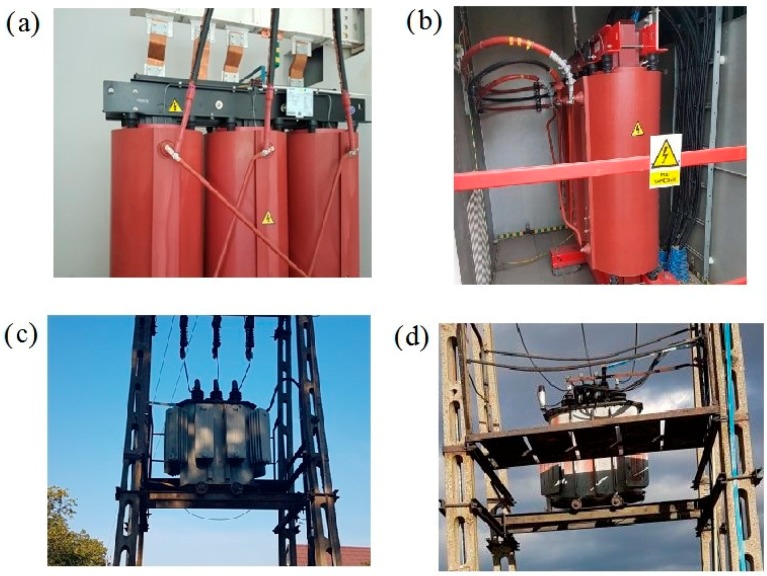
Photos of the tested power transformers: (**a**) first, (**b**) second, (**c**) third, and (**d**) fourth.

The proposed method was tested in a real-world testbed. The measurements of low frequency noise generated by the considered transformers were performed from a single reference point at a distance of 50 m from the sound source. The objects under study were four power transformers with various apparent powers and construction types. A detailed description of the transformers, with the sound pressure level, measured in the full frequency spectrum and narrowed to the low-frequency band, is provided in Table 1. Moreover, measurements were made in two series: firstly, when the transformer was nominally loaded, and secondly, when it was turned off. Figure 4 contains illustrative photos of the tested objects.

**Figure 5:**
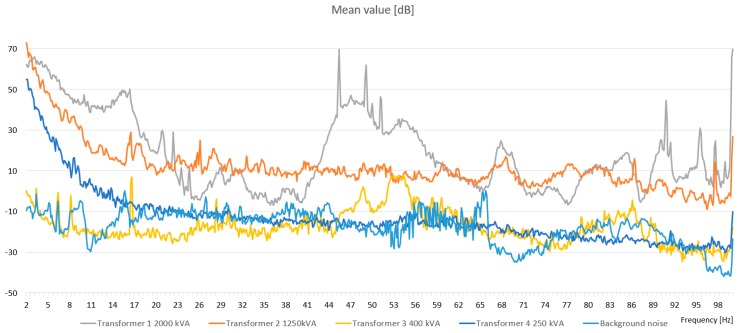
The mean values of transformer noise in the 2–100 Hz frequency range.

**Figure 6:**
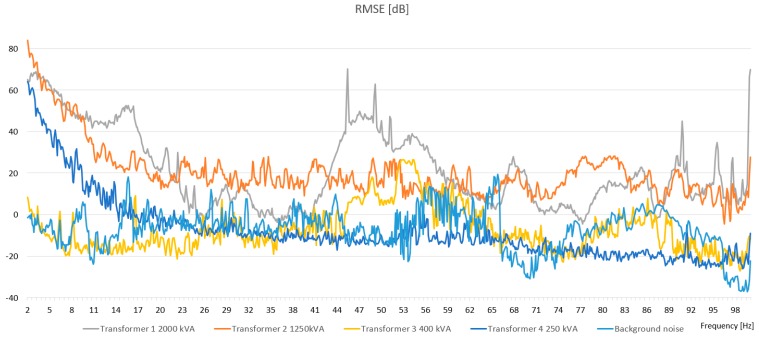
The rms pressure values of transformer noise in the 2–100 Hz frequency range.

The scope of the study includes the analysis of the frequency spectra of sound pressure levels generated during operation by four representative transformers. It is worth noting that the A‑weighted sound level did not exceed 60 dBA in any of the researched transformers. Therefore, the transformers (with measured parameters) were not causing high levels of audible noise. During the research, it was noticed that the rms pressure value changes with the load of the power transformer (especially in low-frequency spectrum). Changes could be observed by relatively high rms pressure values for particular frequencies. The characteristics of the noise were presented as mean and rms values in Figure 5 and Figure 6, respectively.

Figure 5 and Figure 6 presents the frequency spectra of sound pressure levels generated by operating indoor- and overhead-type power transformers for a selected measurement point, i.e., 50 m. In the case of both type of transformers, the determined sound pressure levels of the generated noise were comparable; this applies to the whole range of the investigated frequencies. The largest values of the measured signals were recorded in the infrasound band from 1 Hz to 10 Hz. Several characteristic harmonic components could also be distinguished; the most significant was to be found for an indoor transformer in the range from 7 to 8 Hz, and for an overhead transformer in the range from 14 to 16 Hz. Also, the pick location depended on the transformer type, and was chosen to suit higher frequencies for the overhead transformer. For comparison purposes, the background noise was added in Figure 5 and Figure 6. 

**Figure 7:**
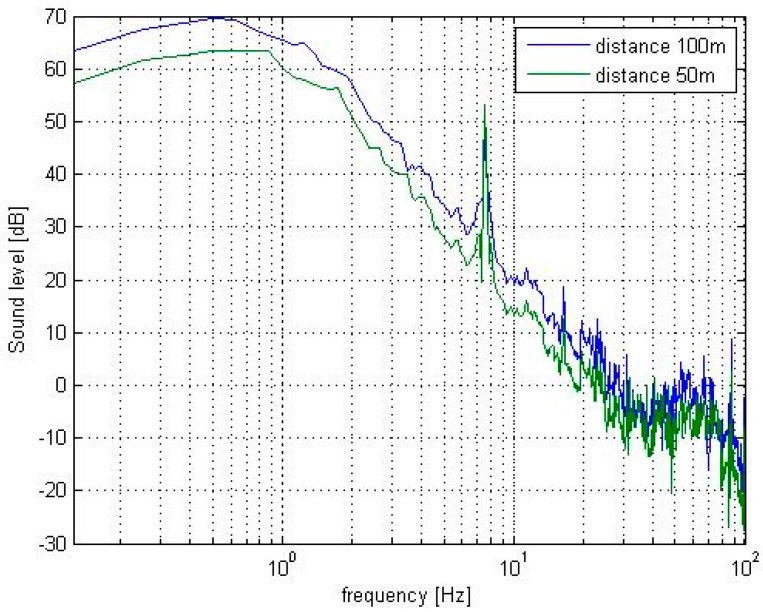
Frequency spectra of sound pressure levels generated during the normal operation of an indoor type power transformer for the selected measurement points.

The tests were also carried out for distances of 75 m and 100 m. The distance did not influence the distribution amplitudes within the analyzed spectra (Figure 7). Additionally, there was a big problem finding objects around which there were no obstacles at a distance of more than 100 m (in the form of houses, trees, or busy roads). Therefore, a distance of 50 m was chosen as optimal for all the tested transformers.

### 4.1. Tuning Method for One Transformer

**Figure 8:**
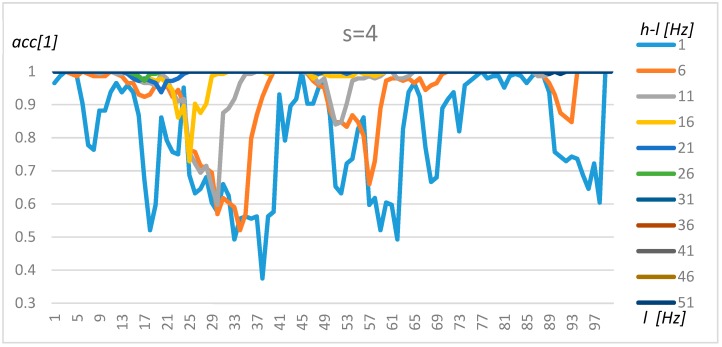
The accuracy value for Transformer 1, s = 4, and various *l* and *h* parameters.

**Figure 9:**
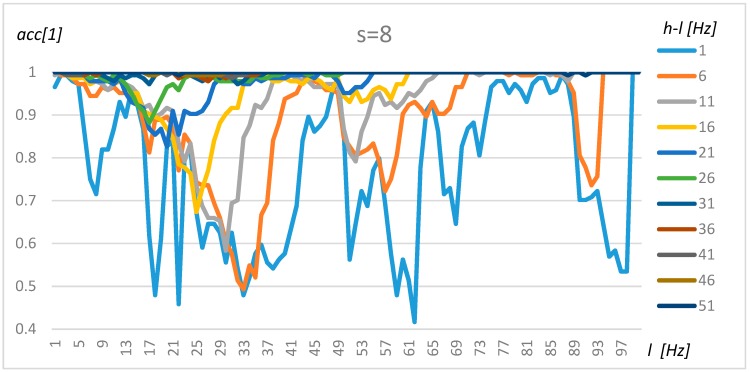
The accuracy value for Transformer 1, s = 8, and various *l* and *h* parameters.

**Figure 10:**
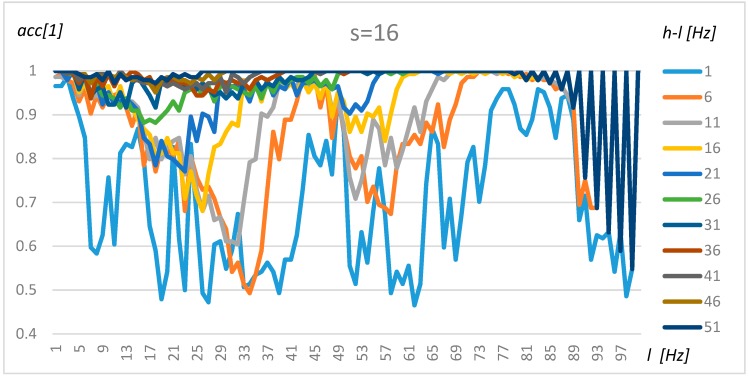
The accuracy value for Transformer 1, s = 16, and various *l* and *h* parameters.

The tuning of the algorithm was performed using the method proposed in Section 3. In the first step, the nominally loaded transformer and its acoustic background were researched based on the resulting data (X). To simplify the tuning procedure and present robust results, the non-parametric, Naïve Bayes and exhaustive method was used. The results for resolution s = 4 (0.5 Hz), s = 8 (1 Hz), and s = 16 (2 Hz) for transformer 1 are presented in Figure 8, Figure 9 and Figure 10, respectively.

An analysis of Figure 8, Figure 9 and Figure 10 demonstrates that the resolution above 1 Hz (s = 8) decreases the stability of the results, which could be observed in Figure 10 as sinusoidal results above 80 Hz. A resolution range of up to 1 Hz gives similar results in terms of accuracy. Furthermore, four frequency ranges can be distinguished which offer a by which manner to classify transformers and their background with a high level of accuracy. The first frequency interval is 2–5 Hz, where the accuracy increases with range and obtains an optimal value for *l* = 2 Hz and *h* = 51 Hz. The second interval could be found at around 40–50 Hz. However, in this case, the accuracy did not increase so rapidly with the range (*h-l*). The third range could be found between 70 Hz and 85 Hz, but gave good results only for *h-l* = 1 Hz and *h-l* = 6 Hz. Finally, a high level of accuracy could be observed for frequencies close to 100 Hz for the smallest range (*h-l* = 1 Hz). A wide frequency range (over 30) makes it possible to obtain an accuracy of above 80% for any frequency. Transformers 2–4 share similar characteristics; however, some intervals were shifted slightly. 

This observation was confirmed by heuristic (non-exhaustive) methods, which tended to find one of those frequency areas. Testing was performed using the Hill climbing method, random searches, and the Bayesian optimization method. The results of the best solution are shown in Table 2.

### 4.2. Tuning Model for Transformer Classification

**Figure 11:**
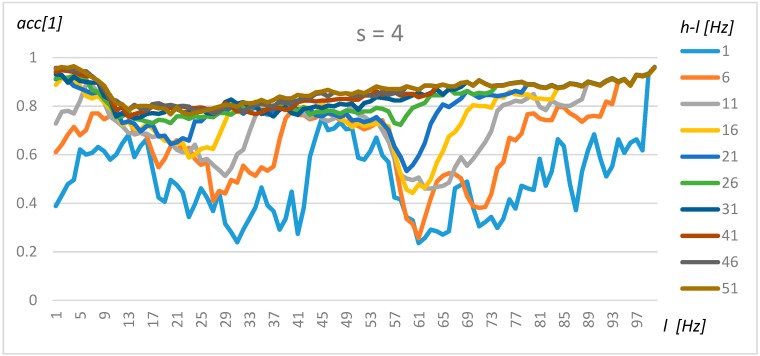
Exhaustive tuning method for resolution 0.5 Hz.

**Figure 12:**
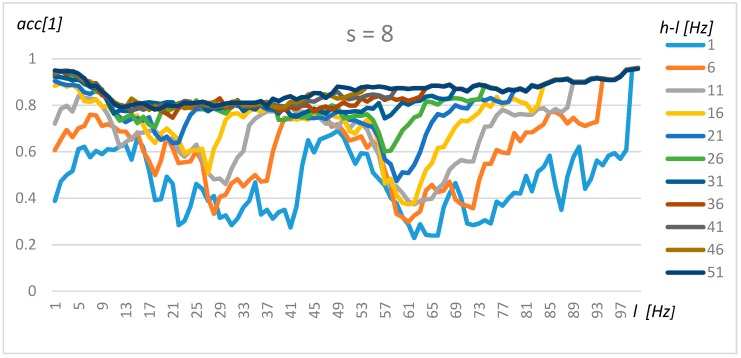
Exhaustive tuning method for resolution 1 Hz.

**Figure 13:**
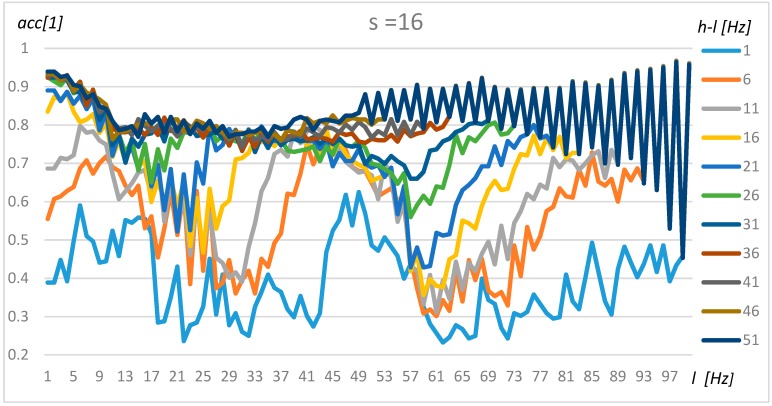
Exhaustive tuning method for resolution 2 Hz.

The tuning of the algorithm for the classification of transformer type was performed in the same manner as for one transformer and its background. The results for resolution s = 4 (0.5 Hz), s = 8 (1 Hz), and s = 16 (2 Hz) are presented in Figure 11, Figure 12 and Figure 13, respectively. 

A comparison of Figure 11, Figure 12 and Figure 13 shows that a resolution of up to s = 8 does not significantly decrease the accuracy. Three frequency ranges can be identified, which makes it possible to classify transformers with a high level of accuracy, i.e., the frequency for *l* = 2 to 5 Hz, where the accuracy increases with range (*h-l* parameter), yielding optimal values for *l* = 2 Hz and *h* = 51 Hz. A second area could be found at around 50 Hz, but for this frequency, the accuracy did not increase equally rapidly with range. Finally, the highest level of accuracy could be observed for frequencies close to 100 Hz. In this case, the small range (*h-l* = 1Hz) was sufficient to obtain a level of high accuracy. The automatically detected frequency intervals yielded comparable levels of accuracy (above 90%).

**Figure 14:**
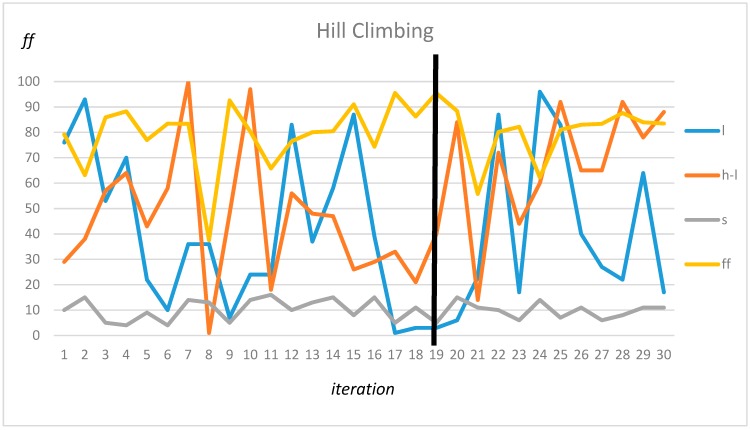
Hill climbing method optimization.

This observation was confirmed by heuristic (non-exhaustive) methods, which yielded the optimal values in those intervals. An example of the Hill Climbing method is presented in Figure 14. In the case of this method, the parameters *l* = 2, *h* = 44, and *s* = 5 were found. The black line illustrates the optimal solution. 

**Figure 15:**
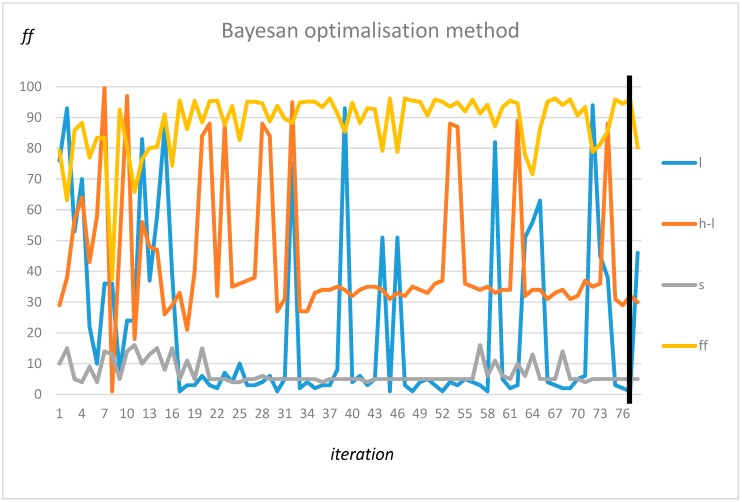
Optimization using Bayesian approach.

Similar results were obtained using a random search: *l* = 4, *h* = 52, and *s* = 5. Finally, the Bayesan optimization method found the same interval as the exhausting method (*l* = 2, *h* = 32, and *s* = 5) parameters. The optimization procedure is presented in Figure 15.

Finally, using calculated parameter *P*, final verification was performed. 

### 4.3. Verification of Tuned Model

Classification was performed for each transformer and its acoustic background. Various optimization methods and machine learning algorithms were tested for this purpose; the results are presented in Table 3 with and without (n/a) preprocessing procedures. In the case when a classifier had additional parameters, their values were tuned as part of the *P* vector.

The research firmly demonstrates that most up-to-date classifiers can obtain an excellent (nearly 100%) classification result and provide automatic identification of transformer noise from the background. The exhaustive search was based on a narrow range close to 1 Hz; thus, in the case of the SVM classifier, the hyperplane was not calculated correctly, and the results were close to 50%. In the case of wider ranges found by heuristic methods (HC and RS), the classifier provided an accuracy level of over 80%. The most stable results were obtained using the Random Trees method. This method also had the highest accuracy without using preprocessing method. The MLP-based solution offered similar results; however, the network tuning process was more complex due to the existence of two parameters to tune (*m*_1_-layer and *m*_2_-neuron number). The proposed preprocessing method made it possible to increase the classification accuracy by 15% on average.

Finally, the transformer type classification was performed for various optimization methods and using various machine learning algorithms. The results are presented in Table 4 using an additional measure, Cohens’ kappa [37].

Similarly, in the case of separate transformers, the SVM method proved to be inefficient (75%) for a small number of attributes. On the other hand, the MLP method, which proved to be a reliable solution for one classifier, provided a lower level of accuracy for the heuristic optimization method. Lower accuracy values were obtained due to fact that the accuracy varied significantly depending on the set of parameters used, and on random factors. Nevertheless, the optimization procedure makes it possible to increase the classification accuracy by 10% on average. The best classification results were obtained using k-NN for the exhaustive search. Nevertheless, most classifiers proved to be able to determine the transformers’ class. Misclassifications, in case of the best result, took place between transformers of the same structure but with different powers.

## 5. Conclusions

The proposed method, using a dedicated sensor, makes it possible to detect the state of transformers (on/off) based on the emitted low frequency with an accuracy of 99%. The best results were obtained using the Random Forest, KNN, and Naïve Bayes methods. The exhaustive search and Bayesian Optimization proved to be the best optimization methods for the transformer low frequency classification problem; however, the heuristic approaches gave better results in cases of separate transformers. 

Further research may be applied to distinguish the type of transformer with a 97% level of accuracy using the KNN method; however, most of the classifiers made it possible to obtain accuracy levels of above 95%. The proposed preprocessing method makes it possible to significantly reduce the number of attributes and increase the detection accuracy by 10% on average. The method, with the proposed preprocessing module, could be adapted for edge computing in nodes due to its simplicity.

The present study has demonstrated that most low frequency noise (information) could be found near the following frequencies: 1Hz, 50Hz, and 100Hz. Therefore, future work will focus on the use multiple frequency ranges to train the classifiers. Furthermore, spectra analyses should be extended to over 100 Hz, due to the fact that vital information can be found near this frequency. This is a first step in future research; in the next stages, we also want to be able to distinguish the power of the transformer and its operating time. This set of parameters can be used for more precise diagnostics.

Finally, the method could be extended to detect noise form unknown transformers and to estimate their type and power level.

## Figures and Tables

**Table 1:** The list of power transformers under study.

Transformer Name	Transformer Type	Apparent Power	Producer	SPL (1–20 kHz) [dB]	SPL (1–100 Hz) [dB]
Transformer 1	indoor type	2000 kVA	Schneider Electric	89	81
Transformer 2	indoor type	1250 kVA	Schneider Electric	89	71
Transformer 3	overhead type	400 kVA	ABB	61	25
Transformer 4	overhead type	250 kVA	ABB	79	49

SPL—sound pressure level.

**Table 2:** The best solutions for heuristic methods.

Resolution [Hz]	Heuristic Method	Start Frequency (*l*)	Range (*h-l*)	Accuracy
0.5	Hill climbing (HC)	38	20	99%
	Random search (RS)	58	28	99%
	Bayesan optimization (TPE)	2	32	99%
1	Hill climbing	67	11	98%
	Random search	89	11	99%
	Bayesan optimization	99	1	99%

**Table 3:** Comparison of various machine learning methods for each transformer and its acoustic background.

Optimal. Method	ML Method	ML Params.	Trans. 1 (Accuracy)	Trans. 2 (Accuracy)	Trans. 3 (Accuracy)	Trans. 4 (Accuracy)
ES	KNN	m_1_ = 5	97%	100%	97%	100%
	naive Bayes	-	97%	100%	100%	97%
	MLP	m_1_ = 1, m_2_ = 8	97%	100%	94%	100%
	RF	m_1_ = 4	100%	100%	94%	100%
	SVM	m_1_ = 0.5, m_2_ = 3	67%	50%	61%	50%
HC	KNN	m_1_ = 5	100%	100%	100%	100%
	naive Bayes	-	100%	100%	100%	100%
	MLP	m_1_ = 1, m_2_ = 8	92%	100%	92%	100%
	RF	m_1_ = 4	100%	100%	100%	100%
	SVM	m_1_ = 0.5, m_2_ = 3	100%	100%	83%	100%
RS	KNN	m_1_ = 5	100%	100%	100%	100%
	naive Bayes	-	100%	100%	100%	100%
	MLP	m_1_ = 1, m_2_ = 8	100%	100%	94%	100%
	RF	m_1_ = 4	100%	100%	100%	100%
	SVM	m_1_ = 0.5, m_2_ = 3	100%	100%	83%	100%
TPE	KNN	m_1_ = 5	100%	100%	100%	97%
	naive Bayes	-	100%	100%	100%	100%
	MLP	m_1_ = 1, m_2_ = 8	97%	97%	100%	92%
	RF	m_1_ = 4	100%	97%	100%	97%
	SVM	m_1_ = 0.5, m_2_ = 3	53%	100%	100%	50%
n/a	KNN	m_1_ = 5	81%	82%	80%	79%
	naive Bayes	-	82%	83%	84%	81%
	MLP	m_1_ = 1, m_2_ = 8	85%	80%	81%	87%
	RF	m_1_ = 4	88%	87%	89%	85%
	SVM	m_1_ = 0.5, m_2_ = 3	84%	82%	86%	88%

EM: exhaustive method; HC: Hill climbing; RS: random search; TPE: Bayesian optimization strategy; KNN: k-Nearest Neighbors; naïve Bayes: Naive Bayes Classification; SVM: Support Vector Machine; RF: Random Forests; MLP: Multiple Layer Perceptron Network.

**Table 4:** Comparison of various machine learning methods.

Optimal. Method	ML Method	ML Params.	Accuracy	Cohens’ Kappa
ES	KNN	m_1_ = 7	97%	96%
	naive Bayes	-	96%	94%
	MLP	m_1_ = 2, m_2_ = 10	93%	91%
	RF	m_1_ = 10	96%	94%
	SVM	m_1_ = 0.5, m_2_ = 3	75%	67%
HC	KNN	m_1_ = 7	94%	93%
	naive Bayes	-	96%	94%
	MLP	m_1_ = 2, m_2_ = 10	86%	81%
	RF	m_1_ = 10	94%	93%
	SVM	m_1_ = 0.5, m_2_ = 3	94%	93%
RS	KNN	m_1_ = 7	94%	93%
	naive Bayes	-	96%	94%
	MLP	m_1_ = 2, m_2_ = 10	86%	81%
	RF	m_1_ = 10	94%	93%
	SVM	m_1_ = 0.5, m_2_ = 3	94%	93%
TPE	KNN	m_1_ = 7	94%	93%
	naive Bayes	-	94%	93%
	MLP	m_1_ = 2, m_2_ = 10	86%	81%
	RF	m_1_ = 10	96%	94%
	SVM	m_1_ = 0.5, m_2_ = 3	96%	94%
n/a	KNN	m_1_ = 7	83%	78%
	naive Bayes	-	86%	81%
	MLP	m_1_ = 2, m_2_ = 10	82%	76%
	RF	m_1_ = 10	85%	80%
	SVM	m_1_ = 0.5, m_2_ = 3	88%	83%

EM: exhaustive method; HC: Hill climbing; RS: random search; TPE: Bayesian optimization strategy; KNN: k-Nearest Neighbors; naïve Bayes: Naive Bayes Classification; SVM: Support Vector Machine; RF: Random Forests; MLP; Multiple Layer Perceptron Network.
